# Prognostic Impact of Cytogenetic Abnormalities in Multiple Myeloma: A Retrospective Analysis of 229 Patients

**DOI:** 10.1097/MD.0000000000003521

**Published:** 2016-05-13

**Authors:** Yuan Jian, Xiaolei Chen, Huixing Zhou, Wanqiu Zhu, Nian Liu, Chuanying Geng, Wenming Chen

**Affiliations:** From the Department of Hematology (YJ, XC, HZ, WZ, NL, CG, WC); and Multiple Myeloma Research Center of Beijing (YJ, XC, HZ, WZ, NL, CG, WC), Beijing Chao-Yang Hospital, Capital Medical University, Beijing, People's Republic of China.

## Abstract

The identification of specific cytogenetic abnormalities by interphase fluorescence in situ hybridization (i-FISH) has become a routine procedure for prognostic stratification of multiple myeloma (MM) patients. In this study, the prognostic significance of cytogenetic abnormalities detected by interphase fluorescence in situ hybridization (iFISH) in 229 newly diagnosed multiple myeloma patients was retrospectively analyzed. Results showed that del (17p), t(4;14), and 1q21 gain were adverse predictors of progression-free survival (PFS). Patients who carried these cytogenetic abnormalities were more likely to have more adverse biological parameters and lower response rate. Multivariate analysis showed that del (17p), t(4;14), and 1q21 gain were statistically independent predictors of PFS, whereas del (17p) was also adverse predictor of overall survival. Multiple coexisting cytogenetic abnormalities also had a negative correlation with PFS. Bortezomib-based therapy could improve the rate and depth of response in patients with t(4;14) translocation and 1q21 gain. Autologous stem cell transplantation could improve, but not overcome the adverse prognostic effect of high-risk cytogenetic abnormalities. These results demonstrate that MM patients with iFISH abnormalities, especially del (17p), are more likely to have a poor prognosis.

## Abstract 2

Supplemental Digital Content is available in the text

## INTRODUCTION

Multiple myeloma (MM) is a heterogeneous plasma cell disorder characterized by genetic abnormalities, including chromosomal translocations, deletions, duplications, and genetic mutations, resulting in a huge heterogeneity in the response rate and survival outcomes.^1,2^ Karyotypes of malignant plasma cells are typically complex, containing numerous numerical and structural abnormalities. Thus, the identification of specific cytogenetic abnormalities by interphase fluorescence in situ hybridization (iFISH) has become a routine procedure for MM prognostic stratification. Hallmark abnormalities include hyperdiploidy, deletion of 17p, gain of 1q21, and translocations involving the immunoglobulin loci. Of these, the aberrations of t(4;14), t(14;16), and 17p deletion [del(17p)] are usually considered to be associated with adverse outcome, whereas the translocation of t(11;14) is associated with relatively better outcome.^2–5^ However, the specific prognostic values of most cytogenetic abnormalities are still controversial.

Nowadays, the treatment strategies have been changed in the era of novel agents. Autologous stem cell transplantation (ASCT) and novel drugs such as proteasome inhibitors (bortezomib) and immunomodulatory drugs (thalidomide and lenalidomide) have become routine therapies in MM. Thus, the prognostic value of iFISH abnormalities in patients receiving different therapies has changed. Previous studies have shown that bortezomib-containing induction regimens improve the outcome of newly diagnosed patients with t(4;14).^6–8^ Therefore the roles of iFISH abnormalities in the prognosis of MM need to be reevaluated. In this study, the prognostic significance of cytogenetic abnormalities detected by iFISH in 229 newly diagnosed multiple myeloma patients were retrospectively analyzed, to provide a comprehensive evaluation of cytogenetic abnormalities.

## PATIENTS AND METHODS

### Patients

A total of 229 newly diagnosed patients with available iFISH results and available treatment information from Beijing Chaoyang Hospital, Capital Medical University (Beijing, China) between August 2010 and November 2014 were included in this study. The diagnostic criteria for symptomatic myeloma used in this study was defined by International Myeloma Working Group.^9^ The International Staging System (ISS)^10^ and Durie-Salmon (DS)^11^ staging system were used to assess patients. A total of 171 of 229 patients received a median of 4 cycles (2–7 cycles, 21 days/cycle) of bortezomib-based regimens, including the basic regimen of bortezomib and dexamethasone (BD), or in combination with doxorubicin (PAD), cyclophosphamide (PCD), thalidomide (PTD), or lenalidomide (PLD). The other 58 patients received a median of 4 cycles (2–8 cycles, 28 days/ cycle) of non-bortezomib-based therapy, involving either thalidomide, doxorubicin, and dexamethasone (TAD), or melphalan, prednisone, and thalidomide (MPT). The responses were evaluated using the International Myeloma Working Group criteria after 4 cycles of induction therapy.^12^ Following the induction therapy, a total of 54 patients received autologous stem cell transplantation (ASCT) and maintenance therapy. The median follow-up time was 24.0 months (range 3.0–73.0 months). The clinical and laboratory features of all 229 patients are summarized in Table 1.

**TABLE 1:**
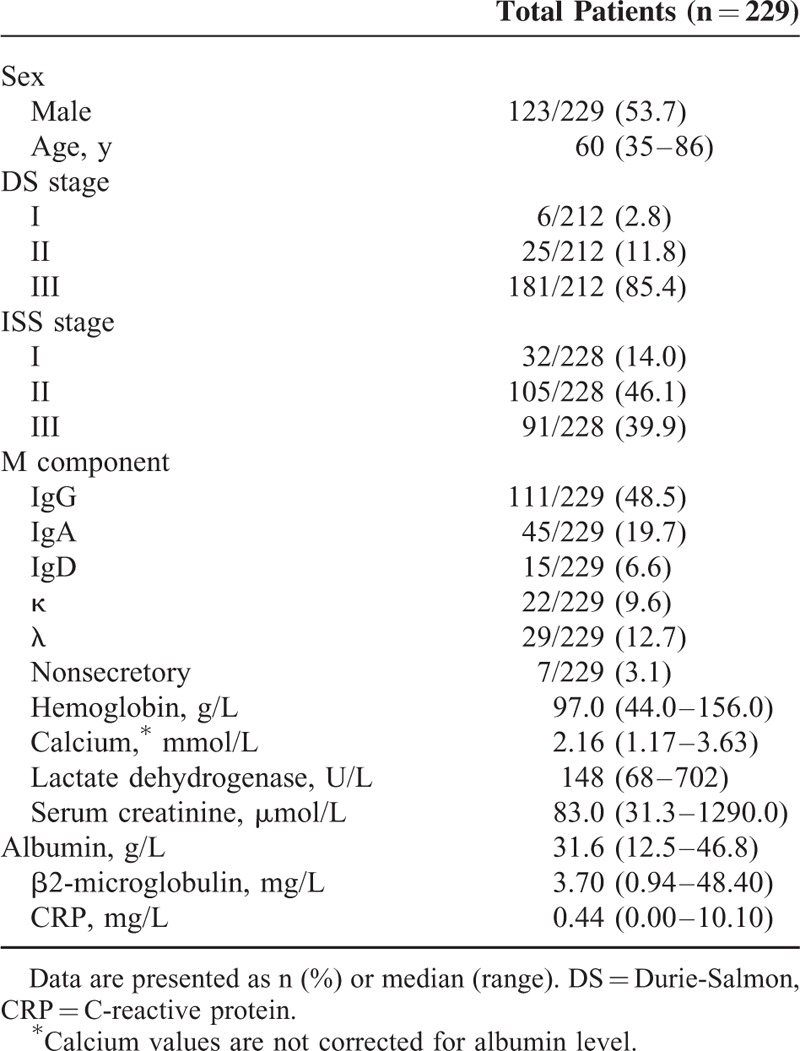
Baseline Patient Characteristics

This study was approved by the ethics committee of Beijing Chaoyang Hospital and written informed consent was obtained from all patients.

### iFISH Analysis

A 5-mL sample of bone marrow was obtained with informed consent from the patients with MM at the time of diagnosis. Mononuclear cells were enriched by the Ficoll-gradient centrifugation method (Ficoll-Paque PLUS; GE Healthcare Bio-Sciences AB, Uppsala, Sweden). Then plasma cells were purified using anti-CD138-coated magnetic beads (Miltenyi technology, Bergisch Gladbach, Germany), enabling a plasma cell purity>90%.^4^ These purified plasma cells were then analyzed using DNA probes (Vysis/ Abbott Molecular, Des Plaines, IL) to detect the following cytogenetic aberrations: del(17p), t(11;14), t(4;14), and t(14;16), as previously reported.^13^ Gains of 1q21 were detected by LSI 1q21 FISH Probe Kit (China Meditech, Beijing, China). A total of 200 interphase nuclei were analyzed. The cutoff value for each iFISH probe was set as >5%.

### Statistical Analysis

Descriptive statistics such as mean, standard deviation, median, and range were used for continuous variables, and frequency counts and percentages were used for categorical variables. Independent sample *t* test was employed to evaluate the associations between genetic abnormalities and biological parameters. *χ*^2^ test or 2-sided Fisher exact test was performed to make comparison of categorical variables among groups. Progression-free survival (PFS) and overall survival (OS) were evaluated according to the international uniform response criteria.^14^ PFS was defined as the duration from the initiation of therapy to the date of death, disease progression, or the last follow-up. OS was defined as the duration from the initiation of therapy to the date of death or last follow-up. Kaplan-Meier method was employed to plot the survival curves, with log-rank test to assess the differences. Cox proportional hazard model for covariate analysis was used to determine the prognostic factors for PFS. All statistical analyses were performed using SPSS version 17.0 (SPSS, Inc, Chicago, IL). The results were considered significant if the *P* value was <0.05.

## RESULTS

### Frequencies and Patterns of Cytogenetic Alterations

Among the 229 patients, all of them were analyzed for del (17p), t(14;16), and t(4;14), and 187 patients were analyzed for t(11;14) and gain of 1q21 additionally. A total of 127 patients of the whole 229 patients (55.5%) exhibited abnormal iFISH results. Whereas when analysis was limited to only those 187 patients who had all the 5 FISH probes tested, abnormalities were found in 64.2% (120/187). These abnormalities were present in del (17p) in 12.7% (29/229), t(14;16) in 1.3% (3/229), t(4;14) in 13.1% (30/229), t(11;14) in 20.3% (38/187), and gain of 1q21 in 43.3% (81/187), respectively. Additionally, 90 of the 229 patients also had results for routine cytogenetic analysis: 10.0% (9/90) were hyperdiploid, 15.6% (14/90) were hypodiploid, 5.6% (5/90) were pseudodiploid, and 68.9% (62/90) were normal karyotype. The relationship between incidence of iFISH abnormalities and patient age was then analyzed. Patients were classified in 3 groups: those younger than 56 years (n = 78), 56 to 65 years (n = 75), and older than 65 years (n = 76). Incidences of the 5 iFISH abnormalities are summarized in Supplementary Table S2. In the 3 groups, statistically significant differences for incidence of total abnormalities and t(11;14) were observed, with lower incidence in older patients. In contrast, incidences of other iFISH abnormalities were not significantly different between the 3 age categories.

The numbers of co-existing iFISH abnormalities were analyzed in the 187 patients who had all 5 FISH probes tested (Table 2). Of these, del (17p) was the most frequently co-existing cytogenetic abnormality, with a probability of 84.0%. Possible correlations between these abnormalities were then analyzed. Results showed a significant correlation between 1q21 gain and del (17p) (frequency of 1q21 gain: 68.0% in patients with del [17p] vs 39.5% in patients lacking del [17p], *P* < 0.01), as well as a significant negative correlation between t(11;14) and t(4;14) (frequency of t(11;14): 0.0% in patients with t(4;14) vs 23.8% in patients lacking t(4;14), *P* < 0.01). Data for t(14;16) were excluded from further analysis because of very small sample size.

**TABLE 2:**
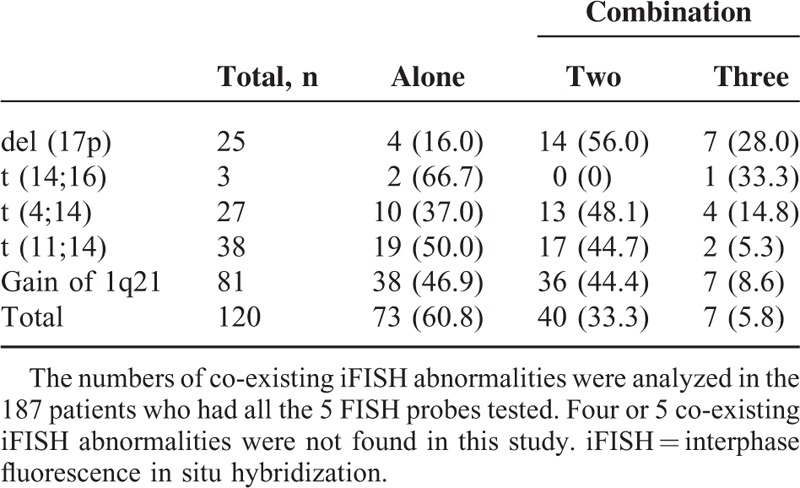
Frequencies of Different Numbers of Co-Existing iFISH Abnormalities

### iFISH Abnormalities and Clinical Characteristics

The correlation between iFISH abnormalities and clinical characteristics was investigated in 229 patients. Those who carried translocation of t(4;14) had lower albumin level (median 28.7 vs 32.4 g/L; *P* < 0.01) and higher β2-microglobulin level (median 3.74 vs 3.62 mg/L; *P* < 0.001) than non-t(4;14) patients, which tended to a higher disease stage and a worse prognosis. Patients carrying gain of 1q21 had significantly lower hemoglobin levels (median 89.0 vs 100.0 g/L; *P* < 0.01) than those without, which also related to adverse biologic characteristics.

### iFISH Abnormalities and Response Rate

A total of 199 patients had available response assessment results after 4 cycles of induction therapy. Among the remaining patients, 14 patients (46.7%) did not receive full induction treatment because of toxicity (3/30, 10.0%), death (2/30, 6.7%), or because they were lost to follow-up before the end of induction therapy (9/30, 30.0%), whereas the others (53.3%) received the full treatment, but did not have formal response assessment at this time point. The overall response rate (ORR) was 89.4% (178/199), including 37.7% complete response (CR), 24.1% very good partial response (VGPR), and 27.6% partial response (PR). Bortezomib-based regimens improved the depth of response in almost all iFISH subgroups (Table 3). The majority did not show statistical significance because of the small sample sizes. Nevertheless, bortezomib treatment resulted in significant response improvements in the del (17p) combination subgroup (at least VGPR: 80.0% vs 20.0%, *P* = 0.03), as well as the gain of 1q21 combination subgroup (at least VGPR: 75.8% vs 25.0%, *P* = 0.01). These data indicated that bortezomib could improve response rate in patients carrying iFISH abnormalities, thus could partially overcome the adverse effect caused by iFISH abnormalities, especially for patients carrying multiple iFISH abnormalities.

**TABLE 3:**
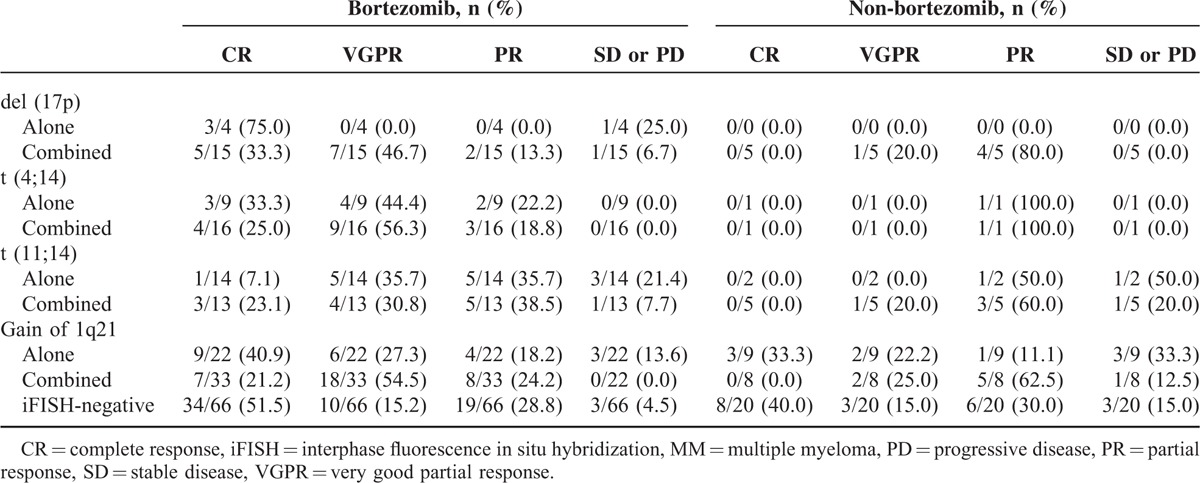
Response Rate of MM Patients With Different iFISH Abnormalities Between Bortezomib- and Non-Bortezomib-Based Therapies

### iFISH Abnormalities and Patient Outcome

The correlation between iFISH abnormalities and patient outcome was then analyzed. Results showed that the abnormalities of del (17p), t(4;14) and 1q21 gain were all as independent adverse prognostic factors of PFS (median PFS times: del (17p) vs non-del (17p): 20.0 vs 35.0 months, *P* < 0.001; t(4;14) vs non- t(4;14): 20.0 vs 33.0 months, *P* < 0.01; 1q21 gain vs non-1q21 gain: 25.0 vs 36.0 months, *P* < 0.001, Table 4]. Although median OS times in all subgroups had not been reached due to short follow-up, del (17p) also was an independent adverse prognostic factor of OS (three-year estimate: 74.2% ± 10.1% vs. 85.9% ± 3.5%, *P* < .01, Table 4). The translocation of t(11;14) was not separately associated with a statistically significant effect on the PFS or OS times.

**TABLE 4:**
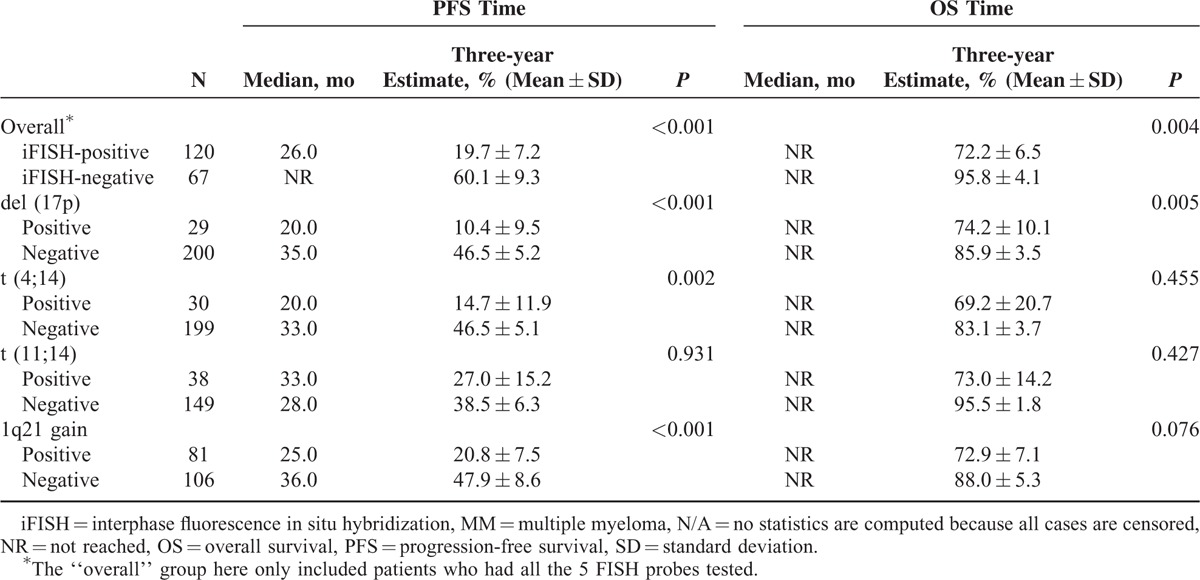
PFS and OS in MM Patients According to Different iFISH Abnormalities

**FIGURE 1:**
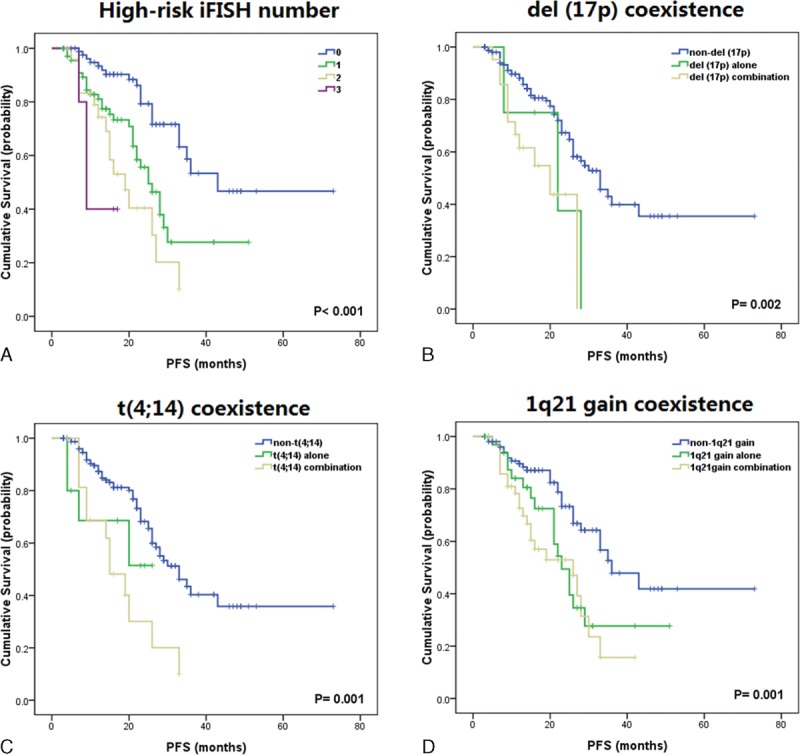
Combinations of cytogenetic abnormalities and PFS. (A) The effect of high-risk iFISH by numbers. (B) Coexistence of del (17p) with other cytogenetic abnormalities. (C) Coexistence of t(4;14) with other cytogenetic abnormalities. (D) Coexistence of 1q21 gain with other cytogenetic abnormalities. iFISH = interphase fluorescence in situ hybridization, PFS = progression-free survival.

The combination effects between different iFISH abnormalities were further analyzed. With the previous results, the three adverse iFISH abnormalities of PFS [del (17p), t(4;14) and 1q21 gain] were considered as high-risk iFISH abnormalities and then were analyzed in combination, whereas those who did not carry any of the 3 iFISH abnormalities were considered as low-risk patients. Results showed that patients who had no high-risk iFISH abnormalities had a median PFS time of 43 months, whereas patients carrying 1, 2, or 3 high-risk iFISH abnormalities had median PFS times of 25, 19, and 9 months, respectively (*P* < 0.001) (Figure 1A), indicating that del (17p), t(4;14), and 1q21 gain could be regarded as risk-stratification factors on patient outcome. When analyzed according to different combination of cytogenetic abnormalities, a strong adverse effect on patient outcome of del (17p), t(4;14), and 1q21 gain combination was found. Median PFS times of del (17p) negative, del (17p) positive alone, and del (17p) combination were 33.0, 22.0, and 20.0 months, respectively (*P* < 0.01) (Figure 1B). The corresponding PFS times of t(4;14) were 33.0, not reached, and 15.0 months, whereas that of 1q21 gain were 36.0, 23.0, and 26.0 months, respectively (Figure 1C, D), indicating that a combination of high-risk iFISH abnormalities was associated with a more adverse prognosis. Because of the limited number of cases, most subgroup analyses did not show statistical significance except for the 1q21 gain group, in which the difference between 1q21 gain negative and 1q21 gain alone (median PFS: 36.0 vs 23.0 months, *P* = 0.01), as well as between 1q21 gain negative and 1q21 gain combination (median PFS: 36.0 vs 26.0 months, *P* < 0.001) were both statistically significant, providing the evidence that 1q21 gain alone could also be seen as an adverse prognostic factor for PFS. The OS data were not shown, as >90% of the data were censored because of short follow-up.

**FIGURE 2:**
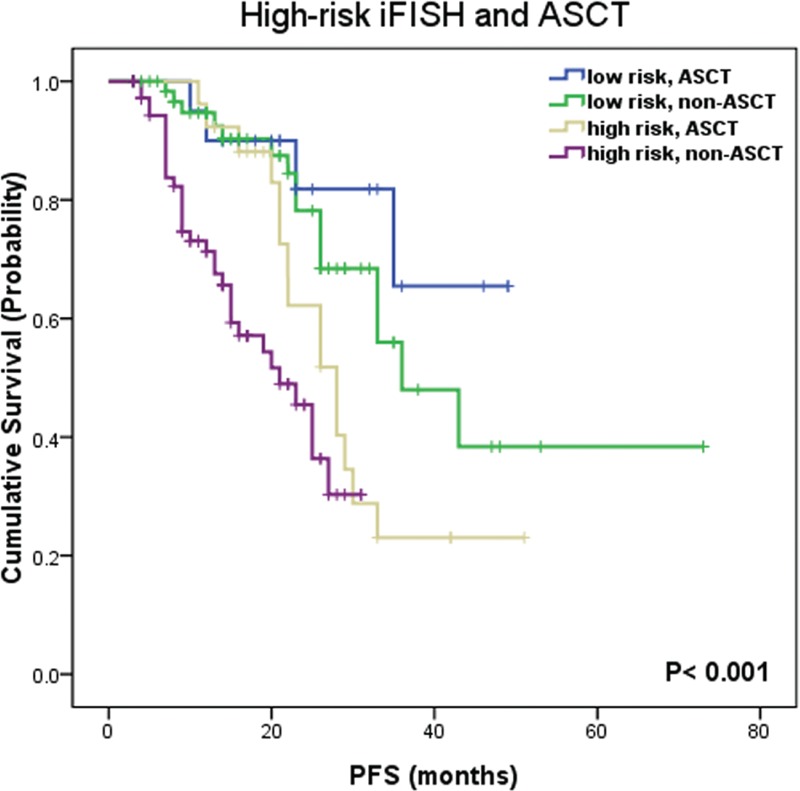
Impact on survival of ASCT and high-risk cytogenetic abnormalities [del (17p), t(4;14) and 1q21 gain]. PFS = progression-free survival, ASCT = autologous stem cell transplantation.

In the 229 patients, a total of 54 patients (23.6%) underwent ASCT. The relationship of ASCT and high-risk iFISH abnormalities [del (17p), t(4;14), and 1q21 gain] was analyzed. Results showed that ASCT prolonged median PFS time of high-risk patients (from 21.0 months to 28.0 months, *P* = 0.08). However, in patients receiving ASCT, presence of high-risk iFISH abnormalities was still an adverse factor on PFS (median PFS time: 28 months in high-risk patients vs not reached in low-risk patients, *P* = 0.03), indicating that ASCT could improve, but not overcome the adverse prognostic effect of high-risk iFISH abnormalities (Figure 2).

### Multivariate Analysis

Univariate and multivariate analysis of PFS was performed on these iFISH abnormalities, as well as other parameters associated with survival in this series. In univariate analyses, several parameters were associated with shorter PFS: del (17p) (*P* < 0.001), t(4;14) (*P* < 0.01), gain of 1q21 (*P* < 0.001), as well as hemoglobin <97 g/L (*P* = 0.01), and β2-microglobulin of ≥3.70 mg/L (*P* = 0.021). After multivariable adjustment for selected clinical and laboratory parameters, the association between iFISH abnormalities and short PFS prevailed. Of these, 17p deletion (hazard ratio [HR] 2.255 [95% confidence interval, CI: 1.146–4.433], *P* = 0.02), t(4;14) (HR 3.415 [95% CI: 1.703–6.848], *P* < 0.01) and gain of 1q21 (HR 1.881 [95% CI: 1.120–3.158], *P* = 0.02) were statistically independent predictors of PFS in multivariate analysis (Table 5). OS analysis was not done because of too many censored data caused by short follow-up.

**TABLE 5:**
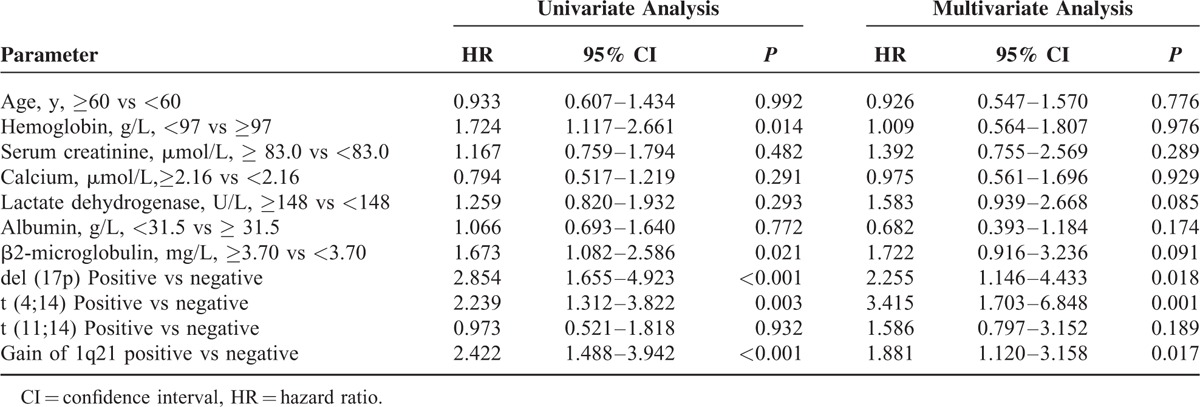
Parameters Associated With Progression-Free Survival

## DISCUSSION

Cytogenetic changes are the hallmark of a wide variety of cancer types, including MM. In this retrospective study, the prognostic impact of cytogenetic aberrations detected by iFISH was analyzed in 229 newly diagnosed Chinese myeloma patients. Our results reveal that del (17p), t(4;14), and 1q21 gain are independent adverse predictors of PFS. Patients carrying these cytogenetic abnormalities are likely to have more adverse biological parameters and lower response rate. The co-existence of iFISH abnormalities relates to more adverse outcome.

With the 5 iFISH probes [del (17p), t(14;16), t(4;14), t(11;14), and gain of 1q21] used in this study, the incidence of each cytogenetic abnormality was 12.7%, 1.3%, 13.1%, 20.3%, and 43.3%, respectively, among which the incidence of t(14;16) in this study is slightly lower than previously reported data in other studies where it has been around 5%, whereas the prevalence of other iFISH abnormalities appears mostly consistent with other published data.^4,15–17^ Furthermore, the incidence of hyperdiploid in this study is only 10.0%, which is lower than previously reported studies in Western population where the incidence is around 50%.^4,16^ Moreover, since patient age is one of the major prognostic factors in myeloma outcome and is associated with incidence of certain cytogenetic abnormalities,^18^ we then analyzed the incidence of cytogenetic abnormalities in different age groups and observed a tendency to lower incidence of iFISH abnormalities in older patients. However, most of the abnormalities did not show statistically significant differences, probably because of small sample sizes. Further studies would be needed to clarify this.

To obtain a comprehensive analysis of the main prognostic parameters, we focused on the most important genetic prognostic factors [ie, del (17p), t(4;14), and 1q21 gain]. As previously shown in many studies,^2,18,19^ del (17p) and t(4;14) remain adverse genetic factors. According to our results, del (17p) is an independent adverse prognostic factor of PFS (20.0 vs 35.0 months, *P* < 0.001) and OS (median not reached, *P* < 0.01). As the most frequently co-existing cytogenetic abnormality, del (17p) exhibits more adverse effect on PFS when in combination with other iFISH abnormalities. As another widely accepted adverse prognostic factor, the translocation t(4;14) also shows adverse prognostic effect on PFS. Furthermore, it is also related to lower serum albumin and higher β2-microglobulin, which tends to a higher disease stage and poorer prognosis, thus providing another interpretation of its adverse effect.

The prognostic value of 1q21 gain has been controversial. Some argue that 1q21 gain does not independently contribute to adverse outcome.^4,20^ On the contrary, a majority of studies consider 1q21 may worsen the prognosis.^13,21–23^ With our present data, gain of 1q21 exerts a marked effect on reduced PFS (25.0 vs 36.0 months, *P* < 0.001). Furthermore, patients carrying 1q21 gain have significantly lower hemoglobin levels than those without 1q21 gain, which also indicates an advanced disease stage. Although 1q21 gain seems to be more frequent in patients with del (17p), 1q21 gain alone is significantly associated with poor outcome, which means that the adverse effect of 1q21 gain could not be simply attributed to the co-existence with other cytogenetic abnormalities. In the prognostic analyses, 1q21 gain is also an independent prognostic parameter for PFS. These data suggest that 1q21 analysis should be added to the diagnostic panel of iFISH probes used in the routine assessment of prognosis in patients with MM. Owing to the limited follow-up time, further follow-up data are needed to better illustrate the prognostic roles of t(11;14) as well as other cytogenetic abnormalities.

As independent adverse prognostic factors, del (17p), t(4;14), and 1q21 gain are considered as high-risk cytogenetic abnormalities in our study. The effect of the numbers of these 3 abnormalities on patient outcome was then analyzed. According to our results, the number of co-existing cytogenetic abnormalities has a negative correlation with PFS times, which means the more high-risk cytogenetic abnormalities, the shorter PFS times of patients. That provides a cytogenetic prognostic model of newly diagnosed MM patients. However, in the multivariate analysis, most of the other adverse factors, such as serum albumin and β2-microglobulin, were nonsignificant, probably because there were insufficient numbers to show a difference.

With the wide administration of ASCT and novel drugs such as the proteasome inhibitor bortezomib in the past decade, the response rate and survival time of MM patients have been improved. Therefore, the role of bortezomib and ASCT in the prognostic impact of iFISH was analyzed in this study. According to our results, bortezomib could improve response rate in patients carrying iFISH abnormalities, thus could partially overcome the adverse effect caused by iFISH abnormalities, especially for patients carrying multiple iFISH abnormalities, also agreeing with previously published reports.^24,25^ As to ASCT, our data showed that ASCT prolonged median PFS time of high-risk patients. However, in patients receiving ASCT, the presence of high-risk iFISH abnormalities was still an adverse factor on PFS, indicating that ASCT could improve, but not overcome the adverse prognostic effect of high-risk iFISH abnormalities. These results suggest that aggressive therapies are still recommended on patients carrying high-risk iFISH abnormalities to ameliorate the negative prognostic value of high-risk cytogenetic abnormalities.^24^

As a retrospective study, this research does have some limitations. One main limitation is the treatment bias of the patients, that is, patients who carried adverse cytogenetic abnormalities were usually administrated more advanced therapies than those with normal iFISH results, which made the analyses more complicated. Another limitation is that not all patients had all the 5 FISH probes tested, and that other cytogenetic abnormalities such as chromosome 1p loss and 13q deletion were not included in this study. The numbers going on to receive ASCT were lower than in other studies because of the financial constraints of patients. In addition, most OS data of these comparisons are not available because of the limited follow-up time. Further follow-ups are needed to investigate a more comprehensive relationship between iFISH abnormalities and outcome.

In conclusion, this study demonstrates that del (17p), t(4;14), and 1q21 gain are independent adverse prognostic factors of MM patients. Those who carry these high-risk abnormalities are more likely to relapse early, thus needing more intensive treatments. iFISH tests can be a valuable risk-stratification factor in prognosis of MM patients.

## Supplementary Material

Supplemental Digital Content

## References

[1] Chng WJ Dispenzieri A Chim CS IMWG consensus on risk stratification in multiple myeloma. *Leukemia* 2014; 28:269–277. 23974982 10.1038/leu.2013.247

[2] Sawyer JR The prognostic significance of cytogenetics and molecular profiling in multiple myeloma. *Cancer Genet* 2011; 204:3–12. 21356186 10.1016/j.cancergencyto.2010.11.002

[3] Walker BA Leone PE Chiecchio L A compendium of myeloma-associated chromosomal copy number abnormalities and their prognostic value. *Blood* 2010; 116:e56–e65. 20616218 10.1182/blood-2010-04-279596

[4] Avet-Loiseau H Attal M Moreau P Genetic abnormalities and survival in multiple myeloma: the experience of the Intergroupe Francophone du Myelome. *Blood* 2007; 109:3489–3495. 17209057 10.1182/blood-2006-08-040410

[5] Inamoto Y Kurahashi S Imahashi N Combinations of cytogenetics and international scoring system can predict poor prognosis in multiple myeloma after high-dose chemotherapy and autologous stem cell transplantation. *Am J Hematol* 2009; 84:283–286. 19338045 10.1002/ajh.21390

[6] Avet-Loiseau H Leleu X Roussel M Bortezomib plus dexamethasone induction improves outcome of patients with t(4;14) myeloma but not outcome of patients with del(17p). *J Clin Oncol* 2010; 28:4630–4634. 20644101 10.1200/JCO.2010.28.3945

[7] Chang H Trieu Y Qi X Bortezomib therapy response is independent of cytogenetic abnormalities in relapsed/refractory multiple myeloma. *Leuk Res* 2007; 31:779–782. 16996589 10.1016/j.leukres.2006.08.002

[8] Pineda-Roman M Zangari M Haessler J Sustained complete remissions in multiple myeloma linked to bortezomib in total therapy 3: comparison with total therapy 2. *Br J Haematol* 2008; 140:625–634. 18302711 10.1111/j.1365-2141.2007.06921.x PMC3655432

[9] International Myeloma Working G. Criteria for the classification of monoclonal gammopathies, multiple myeloma and related disorders: a report of the International Myeloma Working Group. *Br J Haematol* 2003; 121:749–757. 12780789

[10] Greipp PR San Miguel J Durie BG International staging system for multiple myeloma. *J Clin Oncol* 2005; 23:3412–3420. 15809451 10.1200/JCO.2005.04.242

[11] Durie BG Salmon SE A clinical staging system for multiple myeloma. Correlation of measured myeloma cell mass with presenting clinical features, response to treatment, and survival. *Cancer* 1975; 36:842–854. 1182674 10.1002/1097-0142(197509)36:3<842::aid-cncr2820360303>3.0.co;2-u

[12] Rajkumar SV Harousseau JL Durie B Consensus recommendations for the uniform reporting of clinical trials: report of the International Myeloma Workshop Consensus Panel 1. *Blood* 2011; 117:4691–4695. 21292775 10.1182/blood-2010-10-299487 PMC3710442

[13] Liu N Zhou H Yang G Retrospective analysis of genetic abnormalities and survival in 131 patients with multiple myeloma. *Oncol Lett* 2015; 9:930–936. 25624913 10.3892/ol.2014.2750 PMC4301547

[14] Anderson KC Kyle RA Rajkumar SV Clinically relevant end points and new drug approvals for myeloma. *Leukemia* 2008; 22:231–239. 17972944 10.1038/sj.leu.2405016

[15] Fonseca R Blood E Rue M Clinical and biologic implications of recurrent genomic aberrations in myeloma. *Blood* 2003; 101:4569–4575. 12576322 10.1182/blood-2002-10-3017

[16] Nemec P Zemanova Z Kuglik P Complex karyotype and translocation t(4;14) define patients with high-risk newly diagnosed multiple myeloma: results of CMG2002 trial. *Leuk Lymphoma* 2012; 53:920–927. 22023516 10.3109/10428194.2011.634042

[17] An G Li Z Tai YT The impact of clone size on the prognostic value of chromosome aberrations by fluorescence in situ hybridization in multiple myeloma. *Clin Cancer Res* 2015; 21:2148–2156. 25652456 10.1158/1078-0432.CCR-14-2576

[18] Avet-Loiseau H Hulin C Campion L Chromosomal abnormalities are major prognostic factors in elderly patients with multiple myeloma: the intergroupe francophone du myelome experience. *J Clin Oncol* 2013; 31:2806–2809. 23796999 10.1200/JCO.2012.46.2598 PMC3718879

[19] Moreau P Cavo M Sonneveld P Combination of international scoring system 3, high lactate dehydrogenase, and t(4;14) and/or del(17p) identifies patients with multiple myeloma (MM) treated with front-line autologous stem-cell transplantation at high risk of early MM progression-related death. *J Clin Oncol* 2014; 32:2173–2180. 24888806 10.1200/JCO.2013.53.0329 PMC4879712

[20] Fonseca R Van Wier SA Chng WJ Prognostic value of chromosome 1q21 gain by fluorescent in situ hybridization and increase CKS1B expression in myeloma. *Leukemia* 2006; 20:2034–2040. 17024118 10.1038/sj.leu.2404403

[21] Boyd KD Ross FM Chiecchio L A novel prognostic model in myeloma based on co-segregating adverse FISH lesions and the ISS: analysis of patients treated in the MRC Myeloma IX trial. *Leukemia* 2012; 26:349–355. 21836613 10.1038/leu.2011.204 PMC4545515

[22] Hanamura I Stewart JP Huang Y Frequent gain of chromosome band 1q21 in plasma-cell dyscrasias detected by fluorescence in situ hybridization: incidence increases from MGUS to relapsed myeloma and is related to prognosis and disease progression following tandem stem-cell transplantation. *Blood* 2006; 108:1724–1732. 16705089 10.1182/blood-2006-03-009910 PMC1895503

[23] Grzasko N Hus M Pluta A Additional genetic abnormalities significantly worsen poor prognosis associated with 1q21 amplification in multiple myeloma patients. *Hematol Oncol* 2013; 31:41–48. 22674819 10.1002/hon.2018

[24] Kaufman GP Gertz MA Dispenzieri A Impact of cytogenetic classification on outcomes following early high-dose therapy in multiple myeloma. *Leukemia* 2016; 30:633–639. 26487275 10.1038/leu.2015.287

[25] Kalff A Spencer A The t(4;14) translocation and FGFR3 overexpression in multiple myeloma: prognostic implications and current clinical strategies. *Blood Cancer J* 2012; 2:e89. 22961061 10.1038/bcj.2012.37 PMC3461707

