# Supplementary material for: Prognostic Impact of Cytogenetic Abnormalities in Multiple Myeloma: A Retrospective Analysis of 229 Patients
Source: Medicine (Baltimore). 2016 May 13;95(19):e3521. doi: 10.1097/MD.0000000000003521 (PMC4902489; doi:10.1097/MD.0000000000003521)
Supplement: Supplemental Digital Content [file medi-95-e3521-s001.docx]

Jian et al. Prognostic impact of cytogenetic abnormalities in multiple myeloma: a retrospective analysis of 229 patients.

**Supplementary Table S1**. Characteristics of autologous stem cell transplantation patients

|  |  | Total patients (n=54) |
| --- | --- | --- |
| Gender | Male | 30/54 (55.6) |
| Median age at transplantation (years) |  | 51 (35-65) |
| DS stage* | I | 0/49 (0.0) |
|  | II | 7/49 (14.3) |
|  | III | 42/49 (85.7) |
| ISS stage | I | 8/54 (14.8) |
|  | II | 29/54 (53.7) |
|  | III | 17/54 (31.5) |
| M component | IgG | 26/54 (48.1) |
|  | IgA | 13/54 (24.1) |
|  | IgD | 5/54 (9.3) |
|  | κ | 3/54 (5.6) |
|  | λ | 4/54 (7.4) |
|  | Nonsecretory | 3/54 (5.6) |
| Prior treatment | Bortezomib | 40/54 (74.1) |
|  | Non-bortezomib | 14/54 (25.9) |
| Median cycles of induction therapy |  | 4 (3-8) |
| Response before transplantation | CR | 17/54 (31.5) |
|  | VGPR | 18/54 (33.3) |
|  | PR | 18/54 (33.3) |
|  | SD | 1/54 (1.9) |
| Response after transplantation | CR | 28/54 (51.9) |
|  | VGPR | 15/54 (27.8) |
|  | PR | 11/54 (20.4) |
| Data are presented as n (%) or median (range). CR: complete response; VGPR: very good partial response; PR: partial response; SD: stable disease.  * Numbers of DS stage is less due to data missing. | | |

**Supplementary Table S2**. Incidence of cytogenetic abnormalities according to age group.

| Abnormality | Age group (years) | | | P value |
| --- | --- | --- | --- | --- |
|  | < 56 | 56-65 | >65 |  |
| del(17p) | 11/78 (14.1) | 11/75 (14.7) | 7/76 (9.2) | NS |
| t(14;16) | 2/78 (2.6) | 1/75 (1.3) | 0/76 (0.0) | NS |
| t(4;14) | 12/78 (15.4) | 9/75 (12.0) | 9/76 (11.8) | NS |
| t(11;14) | 16/66 (24.2) | 16/59 (27.1) | 6/62 (9.7) | 0.028 |
| Gain of 1q21 | 29/66 (43.9) | 30/59 (50.8) | 22/62 (35.5) | NS |
| Total * | 45/66 (68.2) | 45/59 (76.3) | 30/62 (48.4) | 0.005 |
| * The “total” group here only included patients who had all the 5 FISH probes tested. NS: not significant. | | | | |

**Supplementary Table S2**. Incidence of cytogenetic abnormalities according to age group.

| Abnormality | Age group (years) | | | P value |
| --- | --- | --- | --- | --- |
|  | < 56 | 56-65 | >65 |  |
| del(17p) | 11/78 (14.1) | 11/75 (14.7) | 7/76 (9.2) | NS |
| t(14;16) | 2/78 (2.6) | 1/75 (1.3) | 0/76 (0.0) | NS |
| t(4;14) | 12/78 (15.4) | 9/75 (12.0) | 9/76 (11.8) | NS |
| t(11;14) | 16/66 (24.2) | 16/59 (27.1) | 6/62 (9.7) | 0.028 |
| Gain of 1q21 | 29/66 (43.9) | 30/59 (50.8) | 22/62 (35.5) | NS |
| Total * | 45/66 (68.2) | 45/59 (76.3) | 30/62 (48.4) | 0.005 |
| * The “total” group here only included patients who had all the 5 FISH probes tested. NS: not significant. | | | | |

**Supplementary Table S3**. Treatment choices according to age group.

| Treatment | Age group (years) | | |
| --- | --- | --- | --- |
|  | < 56 | 56-65 | >65 |
| Bortezomib | 64/78 (82.1) | 52/75 (69.3) | 55/76 (72.4) |
| ASCT | 35/78 (44.9) | 19/75 (25.3) | 0/76 (0.0) |
